# Validating the Heat Stress Indices for Using In Heavy Work Activities in Hot and Dry Climates

**Published:** 2016-06-12

**Authors:** Roohalah Hajizadeh, Farideh Golbabaei, Somayeh Farhang Dehghan, Mohammad Hossein Beheshti, Sayed Mohammad Jafari, Fereshteh Taheri

**Affiliations:** ^a^ Occupational Health Research Center, Qom University of Medical Sciences, Qom, Iran; ^b^ Department of Occupational Health, School of Public Health, Tehran University of Medical Sciences, Tehran, Iran; ^c^ Department of Occupational Health, Faculty of Health, Gonabad University of Medical Sciences, Gonabad, Iran; ^d^ Department of Occupational Health Engineering, Faculty of Medicine, Khatam Al-Nabieen University, Kabul, Afghanistan; ^e^ Expert of Occupational Health Research Center, Iran University of Medical Sciences, Tehran, Iran

**Keywords:** Heat stress index, Physiological responses, Environmental parameters, Validation

## Abstract

**Background:** Necessity of evaluating heat stress in the workplace, require validation of indices and selection optimal index. The present study aimed to assess the precision and validity of some heat stress indices and select the optimum index for using in heavy work activities in hot and dry climates.

**Methods:** It carried out on 184 workers from 40 brick kilns workshops in the city of Qom, central Iran (as representative hot and dry climates). After reviewing the working process and evaluation the activity of workers and the type of work, environmental and physiological parameters according to standards recommended by International Organization for Standardization (ISO) including ISO 7243 and ISO 9886 were measured and indices were calculated.

**Results:** Workers engaged in indoor kiln experienced the highest values of natural wet temperature, dry temperature, globe temperature and relative humidity among studied sections (*P*<0.05). Indoor workplaces had the higher levels of all environmental parameters than outdoors (*P*=0.0001), except for air velocity. The wet-bulb globe temperature (WBGT), predicted heat strain (PHS) and heat stress index (HSI) indices had the highest correlation with the physiological parameters. Relationship between WBGT index and carotid artery temperature (r=0.49), skin temperature (r=0.319), and oral temperature (r=0.203) was statistically significant (*P*=0.006).

**Conclusions:** Since WBGT index, as the most applicable index for evaluating heat stress in workplaces is approved by ISO, and due to the positive features of WBGT such as ease of measurement and calculation, and with respect to some limitation in application of HSI; WBGT can be introduced as the most valid empirical index of heat stress in the brick workshops.

## Introduction

Exposure to thermal energy to the extent that it can cause heat stress is part of physical contaminants in workplace that could jeopardize health and safety of workers in the workplace and is statistically remarkable in developing countries^1-3^.

Working in hot processes, especially in hot and dry climates causes increase deep body temperature from normal range because of the imbalance in body temperature and disorders occur due to heat exposure on workers^4^. Some of these disorders include skin rash, extreme tiredness, loss of consciousness, muscle cramps, heart - cardiovascular problems and increase in work-related accidents^5^. In some cases, heat stress can cause the death of workers^6^.

Heat stress can lead to symptoms such as headache, syncope, heat exhaustion and in severe cases neurological disorders and heatstroke^7^. Eighty percent of heatstroke can lead to death^8^. Workers in various occupations such as foundry, construction, and bakery, agriculture and road construction, especially in the hot season prone to heat stress^9^.

Nowadays, on the issues of heat stress management attention has been paid to identify and control of risk factors of heat stress. Therefore, the assessment of thermal stress in the workplace is very important. These assessments can be done using various indices. Most heat stress indices have been obtained through laboratory studies, while the actual conditions in industry are along with vast changes in environmental and physiological variables^10-12^. There is not integrated index that be acceptable in variety of different weather^2, 13^, for this reason, we require to validate the thermal stress indices in order to have a better describe of environmental conditions. Currently, there are several indices to describe the thermal stress in work environments that each of them has advantages and disadvantages ^14^.

Optimum index should be accurate, useful and applicable for a range of environmental and metabolic conditions, and evaluate workers' exposure without interfering with its performance^15^. The body's response to heat stress is known as a strain that can be measure by using physiological parameters^16^ such as body depth temperature (oral temperature, ear tympanic membrane temperature, rectal temperature and urine temperature) and by correlating them with indices can validate various indices and choose the appropriate and efficient index^17,18^.

In this study, Qom City, central Iran due to geographical location as hot and dry climate and also because of the large number of brick kilns workshops (563 workshops) and its activity were selected as representative occupational groups that have heavy activity. Heat received by the workforce in this occupation, with exposure to heat, especially in summer, along with heavy activity is natural 19. For this reason and the above-mentioned reasons, in this study the number of heat stress indices (wet-bulb globe temperature (WBGT), heat stress index (HSI), predicted heat strain (PHS), effective temperature (ET), corrected effective temperature (CET) and discomfort index (DI)) were assessed and validated, and finally, an index determined and introduced as a valid index.

## Methods

This descriptive and analytical study was performed on 184 men at workshops of brick kilns in the city of Qom in the spring and summer of 2013. A short description of the brickmaking process includes tempering (adding water to the clay soil), molding (putting the clay mix into a mold), drying (allowing the molds to dry in the sun), firing (laying out and heating the bricks in kiln)^12^.

According to the standard of ACGIH^20^, work metabolism of workers was classified in heavy job groups. To measuring the dry temperature, globe temperature, wet natural temperature and relative humidity we used calibrated WBGT meter (MK427 JY model, Casella Company). Air velocity was measured by thermal anemometer (YK-2004ah model, Lutron Company). These measurements were carried out, according to the standard ISO 7243^21^, in three areas, namely the height of the head, abdomen and ankle height and at three intervals including 8-10, 10-13 and 16-19during work shift^3,5^.

In addition, measurement of the physiological parameters was done at the same time of measuring environmental parameters‏. Skin temperature was measured by skin thermometer device (TM905 model, LUTRON Manufacturing Co). Ear Carotid artery temperature measured by thermometer (FT4919 model, AEG company in Germany), heart (pulse) rate and systolic and diastolic blood pressure measured by of wrist barometer (DW-701 model, made in China), and oral temperature were measured by oral thermometer simultaneously with measuring environmental parameters in accordance with ISO9886 standard^22^.

Indices were studied by the following methods:

### WBGT Index

To calculate WBGT index in outdoor and indoor environment used equations 1 and 2, respectively ^5^.

WBGT out = 0.7 Tnw + 0.2 Tg + 0.1 Ta Eq.1

WBGT in = 0.7 Tnw + 0.3 Tg Eq.2

Tnw‏: Natural wet temperature

Tg: globe temperature

Ta: dry temperature

In workplace where workers receive heat by process, in addition to environment, assuming that environment is heterogeneous, calculations were performed by Equation 3 according to standard ISO 7243 ^21^.

WBGT= (WBGT_ head_+ (2 × WBGT _abdomen_) + WBGT _feet_) /4 Eq.3

By considering that workers used ordinary working clothes in workplace, correction factor of 0.6 was considered in WBGT calculations.

### DI index

The following equation was used to calculate the index DI^13^;

DI =0.5 T_nw_ + 0.5 Ta Eq.4

T_nw_‏: Natural wet temperature

Ta: dry temperature

### HSI index

This index was calculated by the following equation^23^:

Eq.5 $$HSI=\frac{Ereq}{E\;\max}\times 100$$

In above equation:

Ereq: The thermal energy is required to be excreted from the body by evaporation to achieve thermal equilibrium^23^.

*Ereq = M – R– C* Eq.6

Where‏:

M: Metabolism in *w/m*^2^

R: energy exchanged by radiation and its value (*w/m*^2^) is equal to:

R=4.4 (35-MRT**)** Eq.7

C: energy exchanged by convection and its value (*w/m*^2^) is equal to:

C=4.6V^0.6^ (35-ta) Eq.8

Emax: maximum energy excreted from the body by evaporation in the working environment:

Emax=7V^0.6^ (56-Pa) (w/m2) Eq.9

Pa: pressure of water vapor in air (mb)

V: Air flow rate (*m/s*)

Predicted heat strain) PHS (index):

The software recommended in standard ISO7933^24^ is used to calculate PHS (predicted heat strain).

### CET and ET indices

These indices were derived from the specific diagrams and using of some factors including drying temperature, radiation temperature and wet temperature ^25^. As standard, the TWA (Time Weighting Average) index is calculated for different time using the followingequation^17^:

Index_ TWA_ = (Index_TWA1_×T_1_) + (Index_TWA2_×T_2_) +…. + (Index_TWAn_ ×T_n_)/T_1_+T_2_+…T_n _ Eq.10

For statistical analysis used Microsoft Office Excel 2010 and SPSS 18 software‏.

## Results

The existing tasks at the brick kilns workshop were divided into 4 groups.

1. Those who work on conveyor as conveyors worker.
2. Those who carrying the load (material handling).
3. Those who involved in tempering and molding‏.
4. Those who involved in picking in and picked up brick from the kiln as indoor and outdoor kiln workers.

Number of workers studied in conveyors, handling, tempering and molding and kiln parts are 41 (22.3%), 55 (29.9%), 22 (11.9%) and 66 (35.9%), respectively. Age of workers was between 9 and 70 years and theirs work experience was between 1 to 37 years. According to heat stress criteria recommended by American conference of governmental industrial hygienists (ACGIH) and the metabolic rate of all participants (≈415 w), their work was assigned heavy^2^.

The measurement results of environmental and physiological parameters in various sections are presented in Table 1.Workers engaged in indoor kiln experienced the highest values of natural wet temperature, dry temperature, globe temperature and relative humidity among studied sections (*P*<0.05; one-way ANOVA).As can be seen in Table 1, indoor kiln had the higher levels of all environmental parameters than other sections (*P*=0.0001**)**, except for air velocity. Data related to the physiological responses of the workers shown that mean of oral temperature, skin temperature and ear carotid artery temperature in workers of indoor kiln is higher than ones in other sections (*P*<0.05).

**Table 1:** Mean (SD) of environmental and physiological parameters in different sections

**Variable**	**Material** **handling**	**Kiln**		**Working on** **conveyor**	**Tempering** **and molding**	**Total**
		**Indoor**	**Outdoor**			
**Environmental factors**						
Dry-bulb temperature (ºC)	37.55 (2.25)	44.28 (3.39)	37.29 (2.68)	37.27 (1.14)	35.8(1.85)	39.08 (4.03)
Globe temperature (ºC)	45.25 (1.98)	52.15 (5.56)	45.12 (2.30)	45.01 (1.32)	43.57(2.86)	46.86 (4.68)
Natural wet bulb temperature (ºC)	20.74 (1.67)	25.70 (2.32)	20.66 (2.13)	20.6 (0.86)	19.29 (1.40)	21.88 (2.95)
Relative humidity (%)	17.78 (4.89)	35.61 (13.34)	19.19 (3.67)	17.08 (3.68)	19.14 (2.99)	22.75 (11.09)
Air velocity (m/s)	0.21 (0.159)	0.06 (0.042)	0.32 (0.22)	0.16 (0.03)	0.44 (0.22)	0.2 (0.17)
**Physiological parameters**						
Heart rate (beat/min)	74.98 (10.19)	77.5 (8.85)	75.43 (7.59)	73.56 (9.65)	74.54 (11.50)	75.33 (9.70)
Systolic blood pressure (mmHg)	127.23 (13.28)	129.86 (13.45)	129.85 (10.36)	126.74 (14.83)	125.66 (14.22)	127.88 (13.53)
Diastolic blood pressure (mmHg)	75.58 (12.95)	81.34 (11.08)	83.35 (0.52)	80.85 (9.16)	78.03 (9.55)	79.17 (11.07)
Oral temperature (ºC)	36.38 (0.44)	36.92 (0.34)	36.71 (0.85)	36.23 (0.34)	36.91 (1.01)	36.58 (0.61)
Carotid artery ear temperature (ºC)	36.28 (0.35)	36.83 (0.36)	36.45 (0.46)	36.15 (0.30)	36.36 (0.38)	36.43 (0.44)
Skin temperature (ºC)	35.63 (0.49)	36.35 (0.40)	35.75 (0.62)	35.42 (0.44)	36.27 (0.71)	35.86 (0.63)

Table 2 shows descriptive statistics of heat stress indices on studied workers in different tasks. As can be seen, the average of all indices in section of kiln is more than other tasks.

**Table 2:** Descriptive statistics of heat stress indices in different sections

**Section**	**Wet-bulb globe temperature (WBGT) (°C)**	**Predicted heat strain (PHS) (g/h)**	**Heat stress index (HSI) (%)**	**Discomfort index (DI) (°C)**	**Corrected effective temperature (CET) (°C)**	**Effective temperature (ET) (°C)**
Furnace	
Mean (SD)	30.8 (3.45)	905.91 (226.46)	121.32 (44.8)	33.49 (3.69)	29.02 (0.64)	28.26 (1.54)
Range	12.85	700	151.04	13.45	2.64	5.75
Handling	
Mean (SD)	26.74 (1.42)	623.58 (122.33)	60.39 (11.22)	29.16 (1.85)	28.68 (0.64)	26.69 (1.10)
Range	7.78	660	60.08	10.30	2.81	5.75
Conveyors	
Mean (SD)	26.58 (0.46)	591.95 (93.01)	61.54 (4.61)	28.94 (0.67)	28.83 (0.39)	26.81 (0.53)
Range	1.49	390	16.20	2.31	1.16	1.90
Tempering and molding
Mean (SD)	25.13 (0.99)	555.91 (77.50)	47.09 (8.96)	27.39 (1.47)	27.41 (0.94)	25.01 (1.54)
Range	3.49	300	24.63	3.60	3.16	4.00
Total	
Mean (SD)	27.98 (3.11)	709.29 (217.31)	81.29 (4.11)	30.46 (3.40)	28.69 (0.78)	27.09 (1.59)
Range	13.56	830	151.04	13.45	3.43	5.75

In addition, Table 3 shows the comparison of heat stress indices between indoor and outdoor environment. Average of all indices in indoor are more than outdoor environments (*P*=0.001).

**Table 3:** Comparison of heat stress indices between indoor and outdoor environment

**Variables**	**Indoor**	**Outdoor**	***P*** ** value**
Wet-bulb globe temperature (WBGT) (°C)			0.001
Mean (SD)	31.96 (2.8)	26.42 (1.31)	
Range	11.00	8.48	
Predicted heat strain (PHS) (g/h)			0.001
Mean (SD)	976.73 (191.92)	603.94 (109.79)	
Range	680	670	
Heat stress index (HSI) (%)			0.001
Mean (SD)	140.53 (27.98)	57.95 (10.94)	
Range	102.89	60.08	
Discomfort index (DI) (°C)			0.001
Mean (SD)	34.69 (2.98)	28.80 (1.68)	
Range	11.69	10.30	
Corrected effective temperature (CET) (°C)			0.001
Mean (SD)	29.21 (0.33)	28.49 (0.82)	
Range	1.26	3.43	
Effective temperature (ET) (°C)			0.001
Mean (SD)	28.79 (0.98)	26.43 (1.26)	
Range	3.56	5.75	

The correlation coefficient between heat stress indices and physiological parameters as well as between various indices with each other is shown in Table 4 and 5, respectively. The WBGT, PHS and HSI indices had the highest correlation with the physiological parameters among the other heat stress indices. The correlation coefficient between WBGT and HSI was r=0.93 (*P*<0.001). The highest correlation coefficient was found between WBGT and DI (r=0.981; *P*<0.001).

**Table 4:** Pearson's correlation coefficient between the heat stress indices and physiological parameters

**Variables**	**Oral** **Temperature (°C)**	**Skin** **temperature (°C)**	**Ear carotid artery** **temperature** **(°C)**	**Heart rate** ** (beats/min)**	**Systolic blood** **pressure** **(mmHg)**	**Diastolic blood** **pressure** **(mmHg)**
Wet-bulb globe temperature (WBGT) (°C)
r	0.203	0.319	0.490	0.124	0.053	0.091
*P* value	0.006	0.001	0.001	0.097	0.470	0.220
Predicted heat strain (PHS) (g/h)
r	0.270	0.369	0.487	0.127	0.070	0.090
*P* value	0.001	0.001	0.001	0.080	0.340	0.208
Heat stress index (HSI) (%)
r	0.267	0.388	0.539	0.149	0.073	0.091
*P* value	0.001	0.001	0.001	0.001	0.330	0.223
Discomfort index (DI) (°C)
r	0.156	0.305	0.443	0.126	0.065	0.089
*P* value	0.034	0.001	0.001	0.091	0.380	0.230
Corrected effective temperature (CET) (°C)
r	0.239	0.094	0.155	0.024	0.052	0.120
*P* value	0.001	0.206	0.035	0.743	0.480	0.106
Effective temperature (ET) (°C)
r	0.317	0.115	-0.062	0.103	0.065	0.107
*P* value	0.001	0.118	0.403	0.164	0.384	0.151

**Table 5:** Pearson's correlation coefficient among the heat stress indices

**Variables**	**Wet-bulb globe temperature (°C)**	**Predicted heat strain (g/h)**	**Heat stress index (%)**	**Discomfort index (°C)**	**Effective temperature (°C)**	**Corrected effective temperature (°C)**
**Wet-bulb globe temperature (°C)**
r	1.000	0.930	0.930	0.981	0.860	0.529
*P* value	0.001	0.001	0.001	0.001	0.001	0.001
**Predicted heat strain (g/h)**
r	0.930	1.000	0.906	0.899	0.723	0.533
P value	0.001	0.001	0.001	0.001	0.001	0.001
**Heat stress index (%)**
r	0.930	0.906	1.000	0.892	0.767	0.529
P value	0.001	0.001	0.001	0.001	0.001	0.001
**Discomfort index (°C)**
r	0.981	0.899	0.892	1.000	0.894	0.659
P value	0.001	0.001	0.001	0.001	0.001	0.001
**Effective temperature (°C)**
r	0.860	0.723	0.767	0.894	1.000	0.875
P value	0.001	0.001	0.001	0.001	0.001	0.001
**Corrected effective temperature (°C)**
r	0.646	0.533	0.529	0.659	0.875	1.000
P value	0.001	0.001	0.001	0.001	0.001	0.001

## Discussion

Heat stress on workers in indoor and outdoor environments is the main work-related risk agents, especially in heavy activity and in hot and dry areas such as the city of Qom. Nowadays, various indices are used to evaluate the heat stress that each of them has advantages and disadvantages. Determine optimal index for each workplace, can help to better assessment of environment in terms of thermal stress and therefore be effective to improve health plan.

Results of this study show that all heat stress indices in the indoor environment are more than ones in outdoor that was not consistent with another study^17^. This could be due to lack of engineering and management systems in control of heat stress in understudy workshops. In that study^17^, workers in indoor environments did not receive heat through the process.

According to Table 4 significant relationship was found between the indices, including WBGT, HSI, PHS and DI with deep body temperature (oral temperature, ear carotid artery temperature) and skin temperature. Just HSI index showed a significant correlation with the pulse rate and ET index only with oral temperature and CET index showed significant correlation with oral and ears carotid artery temperatures. A study for comparison of heat stress index include WBGT, SW and DI in hot and humid workplace, found that WBGT and DI Index as well as SWreq that now has become PHS index had significant relationship with deep body temperature and skin temperature, consistent with our results ^13^. However, in a steel industry, the indices of HSI, WBGT, ET and CET did not have a significant relationship with deep temperature and oral temperature^10^. Since these studies are field studies, not controlled and due to difference of thermal stress in various work environments, as well as the possible exposure of workers to other stresses, we cannot make definitive judgment on compliance or non-compliance of these studies.

According to Table 4, there was no significant relationship between the index WBGT, HSI, PHS and DI with systolic and diastolic blood pressure that is not compliance with the study of Golbabaei et al. ^13^. Many factors can affect blood pressure such as nutrition, exercise, medication and lifestyle that in this study, due to lack of control of these risk factors, blood pressure changes (including significant or non-significant changes) and cannot be attributed to weather conditions workplace, but certainly these factors have had an intervening role and perhaps be the reason of differences in the results.

To validate the indices, the considered index should have a strong meaningful relationship with physiological parameters to have the required validity^10^. Clantari et al. performed a study in the steel industry, chose optimum index using the correlation between environmental factors and physiological parameters, and introduced P4SR index as the most reliable index for steel industry ^10^. In addition, Golbabaei et al. with study of comparison between the thermal stress indices and choice of optimal index compared thermal stress indices using correlation between the index and heart rate and select WBGT index as optimum index for hot and humid environment^13^. Falahati et al. examined the validity of WBGT and P4SR indices using deep temperature, and concluded that the WBGT index was more reliable than the P4SR index^17^. Chen et al. investigated thermal stress in steel factory workers, and assessed indices with physiological parameters and showed that skin temperature had the highest correlation with WBGT index, besides SWreq index had the greatest correlation with deep body temperature^26^. Moran et al.^27^and Frank et al.^28^used heart rate and deep body temperature to evaluate heat stress.

In this study, optimum index was chosen by studying the correlation coefficient between the various indices with each other as well as with physiological parameters. However, this study showed that the WBGT and heat stress index (HSI) indices had the highest correlation with other physiological parameters among the other heat stress indices; due to the positive features of WBGT such as ease of measurement and calculation, WBGT can be introduced as the most reliable empirical index for assessing the heat stress in heavy activity. WBGT index, as the most applicable index for evaluating heat stress in workplaces for indoor and outdoor environment has been approved by International Organization for Standardization.

This index has shown a good correlation with environmental factors and physiological parameters 2, 3, and 11. On the other hand, HSI index has relatively high correlation with the physiological parameters and other indices, but according the study of the Di Corleto^29^, values of this index is too exaggerated.

When the airflow rate is equal to zero or close to zero, HSI index estimates more than real; In this case, the results have little value and cannot be evaluated^30^. Therefore, the HSI index can be used under certain circumstances and as a supplement index of WBGT.

PHS index is not a good index for brick burning workplace, because this index is suitable for environments where environmental parameters remain constant and not fluctuate and it is applicable for very detailed assessment. According to the results, the environmental parameters are variable in brick kilns workshops and hence to assess heat stress in this job with heavy activity, use of PHS index is not appropriate.

## Conclusions

There is a suitable correlation between physiological measured parameters and WBGT index, and also, since WBGT index, as the most applicable index for evaluating heat stress in workplaces is approved by ISO, and due to the positive features of WBGT such as ease of measurement and calculation, and also with respect to some limitation in application of HSI; WBGT can be a valid empirical index of heat stress in heavy activities such as brick workshops.

## Acknowledgments

This article is part of a research project Supported by Tehran University of Medical Sciences (Grant no: 22877).

## Conflict of interest statement

All authors declare that there are no conflicts of interest.

## Highlights

- Indoor workplaces (indoor kilns) had the higher levels of most environmental parameters than outdoors.
- Wet-bulb globe temperature (WBGT), predicted heat strain (PHS) and heat stress index (HSI) indices had the highest correlation with the physiological parameters.
- The highest correlation coefficient was found between WBGT and DI (discomfort index(.
- WBGT can be a valid empirical index of heat stress in heavy activities such as brick workshops.
